# Bioactive Compounds in Edible Oils and Their Role in Oxidative Stress and Inflammation

**DOI:** 10.3389/fphys.2021.659551

**Published:** 2021-04-30

**Authors:** Alessandra Mazzocchi, Valentina De Cosmi, Patrizia Risé, Gregorio Paolo Milani, Stefano Turolo, Marie-Louise Syrén, Angelo Sala, Carlo Agostoni

**Affiliations:** ^1^Department of Clinical Sciences and Community Health, University of Milan, Milan, Italy; ^2^Pediatric Intermediate Care Unit, Fondazione IRCCS Ca’ Granda Ospedale Maggiore Policlinico, Milan, Italy; ^3^Department of Pharmaceutical Sciences, University of Milan, Milan, Italy; ^4^Pediatric Unit, Fondazione IRCCS Ca’ Granda Ospedale Maggiore Policlinico, Milan, Italy; ^5^Pediatric Nephrology, Dialysis and Transplant Unit, Fondazione IRCCS Ca’ Granda Ospedale Maggiore Policlinico, Milan, Italy; ^6^Istituto per la Ricerca e l'Innovazione Biomedica (IRIB), Consiglio Nazionale delle Ricerche (CNR), Palermo, Italy

**Keywords:** edible oil, vegetable oil, linoleic acid, alpha-linolenic acid, docosahexaenoic acid, immune response, inflammation resolution, marine oil

## Abstract

Diet and inflammatory response are recognized as strictly related, and interest in exploring the potential of edible fats and oils for health and chronic diseases is emerging worldwide. Polyunsaturated fatty acids (PUFAs) present in fish oil (FO), such as eicosapentaenoic acid (EPA) and docosahexaenoic acid (DHA), may be partly converted into oxygenated bioactive lipids with anti-inflammatory and/or pro-resolving activities. Moreover, the co-presence of phenolic compounds and vitamins in edible oils may prevent the development of chronic diseases by their anti-inflammatory, antioxidant, neuroprotective, and immunomodulatory activities. Finally, a high content in mono-unsaturated fatty acids may improve the serum lipid profile and decrease the alterations caused by the oxidized low-density lipoproteins and free radicals. The present review aims to highlight the role of lipids and other bioactive compounds contained in edible oils on oxidative stress and inflammation, focusing on critical and controversial issues that recently emerged, and pointing to the opposing role often played by edible oils components and their oxidized metabolites.

## Introduction

**Figure 1:**
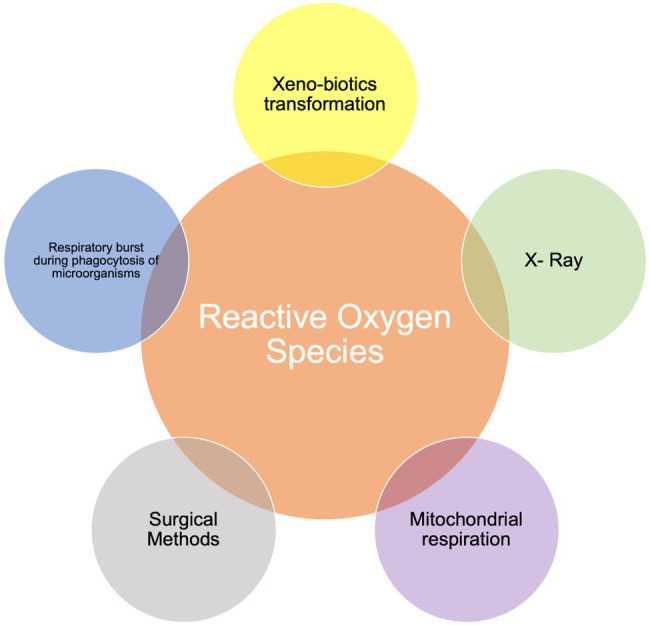
Possible sources of Reactive Oxygen Species (ROS).

Reactive oxygen species (ROS) are radical and non-radical chemical species formed by the partial reduction of oxygen that physiologically accumulate in parallel with cellular aerobic respiration (Angelova and Abramov, 2016). If unchecked, these compounds may result in DNA damages and cellular death. Figure 1 shows possible endogenous and exogenous sources of ROS, highlighting respiration as the major contributor to endogenous ROS production. Moreover, during stress conditions, the endoplasmic reticulum releases Ca^2+^ that may (a) contribute to the activation of the cytoplasmic protein NLR-Family Pyrin Domain Containing 3 (NLRP3) and therefore of the inflammasome, and (b) enter the mitochondria with subsequent generation and release of ROS (Zorov et al., 2014). Cytoplasmic ROS can also activate nuclear transcription factor kappa B (NF-κB), that migrates into the cellular nucleus promoting the transcription of inflammatory and oxidative genes, like cyclooxygenase (COX)-2, inducible-nitric oxide synthases (NOS), tumor necrosis factor alpha (TNF-α), interleukin (IL)-6, and IL-1β (Mitchell et al., 2016). Transcription of inflammatory and oxidative genes can also be activated by Activator Protein 1 (AP-1) and Mitogen-Activated Protein Kinase (MAPK) resulting from toll like receptor’s (TLR) engagement (He et al., 2009; Kuriakose et al., 2019).

Additional sources of ROS include drug-derived ROS produced as a consequence of their oxidative metabolism, X-rays, and surgical interventions, but ROS formation is also significantly associated with the inflammatory response. Inflammation is the physiological response to damage and is normally temporarily limited and solved by either specific or non-specific immune mechanisms. When an inflammatory response to an insult, whichever the origin, is not contained and then eliminated by the immune response, the self-perpetuation extends beyond the primary *foci* within a generalized hyper-reaction generating ROS that may contribute to the pathogenesis of non-communicable chronic diseases (NCDs), including cardiovascular diseases (CVDs), metabolic diseases, and cancer (Liguori et al., 2018).

The body possesses defense mechanisms against ROS, such as specific enzymes (i.e., superoxide dismutase, catalase, and glutathione peroxidase) and thiolic antioxidant (i.e., glutathione, and albumin), and generates biologically active metabolites playing an important role in the physiological resolution of the inflammatory process. The diet may contribute to these processes by providing micronutrients, such as vitamin C and vitamins A and E, that can neutralize ROS, as well as macronutrients such as omega (ω)3 fatty acids that are substrates for the biosynthesis of resolution mediators.

Besides vitamins, other micronutrients can modulate inflammation, including minerals like Se, Cu, and Zn (Galland, 2010), while focusing on macronutrients (that is, dietary components supplying energy, such as proteins, carbohydrates, and fats), hyper-caloric Western diets based on energy-dense foods, rich in simple sugars, and low in fibers, greatly contribute to an increase of endogenous lipogenesis to store the excess of energy. This process leads to high serum levels of saturated fatty acids (SFAs) which, in turn positively correlate with inflammatory markers such as circulating fibrinogen (Galland, 2010). By contrast, consumption of polyunsaturated fatty acids (PUFAs) and ω3 fatty acids in particular, increases their circulating levels and shows opposite associations (He et al., 2009). Compared with ω3, ω6 fatty acids show variable effects on inflammation, and available data are controversial (Innes and Calder, 2018), but dietary, circulating monounsaturated fatty acids (MUFAs), especially oleic acid, may have anti-inflammatory effects (Mashek and Wu, 2015).

Thus, dietary patterns, according to their specific nutrient and food composition, can either help to preserve a functional health status, or increase the risk of developing NCDs. The Mediterranean Diet (MD) is considered a healthy food pattern, especially considering its potential role in protecting against inflammation (Gotsis et al., 2015). The term “Mediterranean Diet” is usually referred to as a diet characterized by the high consumption of fruits, vegetables, whole grain cereals, seafood, legumes, nuts, and seeds, with a limited intake of meat and fermented beverages (Widmer et al., 2015). Replacing the intake of SFAs with PUFAs is recommended by dietary guidelines focused on cardiovascular health [National Cholesterol Education Program (NCEP) Expert Panel on Detection, Evaluation, and T. of H. B. C. in A. (Adult T. P. I.), 2002], and consuming dietary oils derived from plants, seeds, or of marine origin is a strategy to increase the intake of PUFAs.

Plant-derived oils contain the two precursors of the ω6 and ω3 families, i.e., linoleic and alpha-linolenic acids (LA, ALA), together with protective micronutrients, such as tocopherols, carotenoids, phytosterol, beta-carotene, nitrogen compounds, minerals (e.g., phosphorous, magnesium, manganese, copper, iron, zinc, and potassium), vitamins, and phenolic compounds (Vergallo, 2020). Marine-derived oils, in particular oils from fatty fish, are an important source of ω3 fatty acids, predominantly eicosapentaenoic acid (EPA) and docosahexaenoic acid (DHA; Abedi and Sahari, 2014). Freshwater fish also ensures a good supply of ω6 PUFAs, but the possible presence of toxic contaminants or heavy metals and antibiotics may raise concerns in using fish oil (FO) as a source of PUFAs. Furthermore, undesirable flavors and tastes of FO may contribute to limiting their consumption (Abedi and Sahari, 2014).

In the first part of this narrative review, we will focus on the specific families of compounds present in plant- and marine-derived oils and their potential role relative to the formation of ROS and the inflammatory response. Particular attention will be on their specific activity and function, their dietary sources and the principal evidence related to their association with human health.

The second part of this work will briefly summarize a selection of dietary oils (vegetable or marine-derived) focusing on the results of human trials available in the literature highlighting their effects in different disease conditions.

A specific attention will be paid to controversial or debated issues relative to selected components or edible oils.

## Lipids, Lipid-Derived Compounds, and Micronutrients From Edible Oils: Role in Oxidative Stress and Inflammation

Edible oils and their components can play different and opposing roles in oxidative stress and inflammation. During the inflammatory responses and in presence of pathologies characterized by high tissue production of ROS, an increase intake of oils with significant amounts of long-chain PUFAs (LC-PUFAs) may increase PUFA in membrane phospholipids and, because of their high susceptibility to peroxidation (Pamplona, 2008), this could cause increased levels of PUFA peroxidation-derived compounds, such as oxidized phospholipids and isoprostanes, possessing significant pro-inflammatory properties (Takahashi et al., 1992; Marathe et al., 2001; Leitinger, 2003). On the other hand, recent reports identified oxidized phospholipids endowed with potent activities leading to the physiological resolution of the inflammatory process (Friedli and Freigang, 2017), pointing to the balance between these potential effects of LC-PUFA peroxidation as a critical factor in defining the outcome of the increased consumption of LC-PUFAs.

Oxygenase enzymes, such as lipoxygenases and COXs, acting on LC-PUFAs can also contribute to the formation of ROS, both directly, as a by-product of their enzymatic activity (Swindle et al., 2007), and through the formation of ω6 arachidonic acid (AA) metabolites such as leukotriene (LT) B_4_, that activate the NADPH oxidase (NOX; Yun et al., 2010). Conversely, lipoxygenase-derived metabolites of ω3 LC-PUFAs may limit the formation of ROS (Chattopadhyay et al., 2017), control the inflammatory response, and promote its resolution (Serhan et al., 2008), suggesting again how a fine balance may define the contribution of LC-PUFAs to the physiological resolution of the inflammatory response rather than its evolution into chronic inflammation and pathology.

Finally, micronutrients from vegetable oils, such as vitamins or polyphenols, may also provide protection against ROS formation and its effects by their anti-oxidant activity, thus preserving membrane integrity (Ayala et al., 2014; Bochkov et al., 2017) and limiting the formation of pro-inflammatory mediators (Santus et al., 2005). Nevertheless, also in this case, the discussion is ongoing about the ability of these compounds to significantly affect human health in consideration of the low concentrations observed in many edible oils and the limited bioavailability of phenolic compounds (Nediani et al., 2019).

### LC-PUFAs

Long-chain polyunsaturated fatty acids are synthesized by elongation and desaturation of the carbon chain from the parent essential PUFAs: LA for the ω6 series and ALA for the ω3 series (Agostoni and Bruzzese, 1992). The metabolic pathway consists of successive carbon chain elongation and desaturation steps (by inserting double bonds into the carbon chain), that are controlled by elongase and desaturase enzymes, respectively. It begins with a ∆6-desaturation step, followed by chain elongation and desaturation thereof, to yield EPA when the initial substrate is ALA, or to yield AA when the initial substrate is LA (Zárate et al., 2017).

#### Arachidonic Acid and Its Metabolites

Arachidonic acid (AA) is the main ω6 product and is present esterified to the 2-position in specific classes of membrane phospholipids (Chilton and Murphy, 1986). Upon release from membrane phospholipids by the activity of the cytosolic phospholipase A2, AA is enzymatically converted by several oxygenases into eicosanoids, a large family of mostly pro-inflammatory molecules, while non-enxymatic peroxidation of esterified AA leads to the formation of isoprostanes (Morrow et al., 1990), that can be released from phospholipids by the activity of platelet-activating factor acetylhydrolases and soluble phospholipases A2 (Kuksis and Pruzanski, 2017).

Cyclooxygenase (COX)-1 and COX-2 drive the synthesis of prostaglandins (PGs), prostacyclin, and thromboxane (TX; Smith et al., 1996); although these bioactive lipids are involved in a number of physiological and homeostatic processes, including hemostasis, vascular function, and gastric cytoprotection (Robert, 1979; Bunting et al., 1983; Weksler, 1984), they are mostly renowned for their ability to initiate and maintain inflammation. PGD_2_, PGE_2_, PGI_2_, and PGF_2α_ represent to date a central subject of study among eicosanoids in inflammation, especially in light of the ability of NSAIDs to block their synthesis by inhibition of COX-1/2, which in turn results in the prevention of inflammation (Vane, 2002). A significant body of evidence is available that COX-2-derived PGE_2_ may play a role in tumor angiogenesis by increasing vascular endothelial growth factor (VEGF; Eibl et al., 2003), and recent data strongly suggest a contribution of platelet COX-1-derived thromboxane in colorectal cancer (Patrignani and Patrono, 2018).

Non enzymatic isoprostanes have been widely used as marker of oxidative stress *in vivo*, but biological activities through the interaction with the thromboxane receptor have also been reported for 8-isoprostaglandin F_2α_ (Takahashi et al., 1992), suggesting that isoprostanes could play a role in the control of vascular tone and hemostasis (Capra et al., 2014). Recent reports have nevertheless shown that non enzymatic lipid peroxidation of esterified AA also generates cyclopentenone-containing oxydised phospholipids and related isoprostanes possessing potent pro-resolution activities. These compounds inhibit TLR activation and NF-kB signaling, and activate the Nrf2-pathway leading to the expression of anti-oxidant genes, limiting inflammation and cellular damage (Friedli and Freigang, 2017).

5-, 12-, and 15-lipoxygenases (5/12/15-LOX) generate leukotrienes (LTs), hydroxyeicosatetraenoic acids (HETEs; Kuhn et al., 2015), and lipoxins (LXs; Romano et al., 2015); Leukotrienes also play a significant role in inflammation (Sala et al., 1998), and their increased expression has been reported in response to Th2 cytokines; neutrophilic tissue infiltration, and activation in response to LTB4, a main metabolite of 5-LO, is a mainstay of the acute inflammatory response (Mashima and Okuyama, 2015). Again, together with the proinflammatory role of lipoxygenase products, evidence have emerged early on that trihydroxy-derivatives of AA such as lipoxin A4 (LXA4) and LXB4, acting through the G-protein coupled receptor ALX/FPR2, inhibit inflammation responses by leukocytes, endothelial and epithelial cells (Romano et al., 2015).

Finally, diHETEs and epoxyeicosatrienoic acids represent the products of P450 epoxygenases (Bellien and Joannides, 2013), while ω and ω-1 monohydroxy metabolites of AA are generated by P450 ω-hydroxylases. Also in this case while the latter compounds can play pathophysiological role in cancer progression by promoting angiogenesis (Johnson et al., 2015), the former compounds have been reported to be endowed of significant anti-inflammatory activities, mediated by the inhibition of NF-kB, and the increase of peroxisome proliferator-activated receptor-gamma (PPAR-γ) transcription activity (Norwood et al., 2010).

#### ω3 Long Chain PUFAs and Their Metabolites

**Figure 2:**
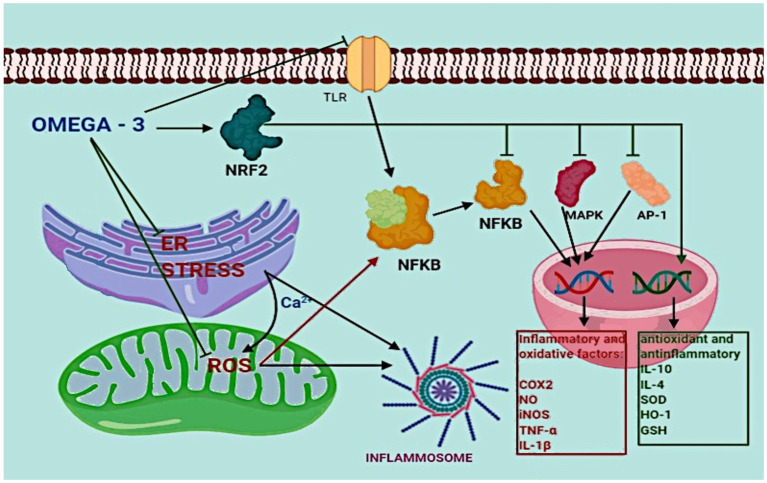
The role of ω-3 fatty acids in inflammation.

The main activities of ω3 LC-PUFAs are to directly and indirectly inhibit the inflammatory response: DHA can reduce endoplasmic reticulum stress and ROS production in mitochondria, inhibit TLR activation, and upregulate cytoprotective proteins, intracellular antioxidants, and anti-inflammatory and detoxifying enzymes through the activation of nuclear factor erythroid 2-related factor 2 (NRF2). Activated NRF2 inhibits the activity of AP-1, NF-κB, and MPK and promotes the transcription of anti-inflammatory and anti-oxidative genes like IL-10, IL-4, superoxide dismutase, heme oxidase-1, and glutathione (Yamagata, 2017). DHA and EPA can regulate the expression of oxidized low-density lipoprotein receptor 1, plasminogen activator inhibitor 1, thromboxane A2 receptor, vascular cell adhesion molecule-1, monocyte chemoattractant protein-1, and intercellular adhesion molecule 1, regulating, de facto, the inflammation response (Yamagata, 2017). Moreover, DHA inhibits TLR activation acting as an antagonist of SFA and blocking the inflammation triggered by TLR (Hwang et al., 2016; Figure 2).

ω3 LC-PUFAs, such as EPA, DPA, and DHA, are also the precursors of a number of oxygenated lipids, typically resulting from the coordinated activities of multiple lipoxygenases, including the 15/17R-lipoxygenase activity resulting from the aspirin acetylation of COX-2 (Serhan et al., 2002). The resulting compounds have been collectively described as a novel genus of specialized pro-resolving lipid mediators (SPM; Serhan et al., 2020) and are endowed with potent anti-inflammatory, pro-resolution activities within the immune system. SPMs, including D- and E-series resolvins, protectins, maresins (Serhan, 2017), and maresin conjugates in tissue regeneration (MCTRs; Dalli et al., 2016; Chiang et al., 2018), proved effective in limiting inflammation and contributing to speed-up the physiological resolution of the inflammatory response (Serhan and Levy, 2018). Several reports have linked supplementation with ω3 LC-PUFAs with increased concentrations of SPM in plasma (Grenon et al., 2015; Mas et al., 2016), but a critical study carried out both using ω3 LC-PUFAs supplementation and LPS challenge *in vivo* in humans raised questions about the effective correlation between plasma SPM and dietary supplementation (Skarke et al., 2015). Recent results using human hepatic cells showed extremely rapid β-oxidation of protectin D1, suggesting that plasma determination of this SPM may not be the appropriate approach to the assessment of its *in vivo* production (Balas et al., 2019). Indeed, *in vivo* production of eicosanoids has always been assessed either at the local site of production (if possible) or through the determination of stable hepatic metabolites in urines (Catella et al., 1986), while plasma concentrations were early on ruled out as unreliable or undetectable. In agreement with these considerations, supplementation with DHA in cystic fibrosis patients resulted in increased concentrations of DHA-derived oxygenated metabolites in sputum (Teopompi et al., 2019), but the relevance of ω3 LC-PUFAs supplementation to the production of SPM remains to be assessed.

### Linoleic Acid and Its Metabolites

Linoleic acid, defined as an essential fatty acid in mammals because of their inability to synthesize it (Marangoni et al., 2020), is common in the human diet, being widely distributed in foods. In many vegetable oils, it represents more than 50% of the lipid content; high amounts of LA are also present in nuts, while lower levels are found in cereals (more in whole grains), legumes, some meats, eggs, and dairy products (Marangoni et al., 2020). In animal cells can be converted into AA; however, LA conversion to AA is very low (< 1%), and increasing the intake of dietary LA does not lead to a significant increase of its metabolites tissue levels (Rett and Whelan, 2011; Whelan and Fritsche, 2013). The effect of LA on human health is still controversial: a recent review discussed evidence about the potential benefit of increasing dietary intake of LA (Marangoni et al., 2020), reporting that epidemiological studies indicate that an adequate intake of LA reduces amounts of plasma low-density lipoprotein cholesterol (LDL-C) and dietary intervention studies showed that replacing 5% of the dietary energy derived from SFA with ω6 PUFAs reduces LDL-C by up to 10% (Siri-Tarino et al., 2015). Furthermore, in prospective cohort study circulating concentrations of LA are inversely associated with new cases of type 2 diabetes (Fretts et al., 2019). On the contrary, the reevaluation of data obtained in double-blind randomized controlled trials (RCTs), together with a systematic review and meta-analysis, while reporting a significant reduction of serum cholesterol did not confirm the original hypothesis of a significant effect on the risk of death by coronary heart disease (CHD) or all causes (Ramsden et al., 2013, 2016). The controversial role of linoleic acid is also underlined by several evidence suggesting that high intake of this PUFA is associated to an increase risk of colonic inflammation and colonic cancer. Indeed the risk of colorectal adenoma increased in correlation to plasma concentrations of linoleic acid in an endoscopy-based case–control study (Pot et al., 2008), and dietary linoleic acid was found to potentially contribute to an increased risk of ulcerative colitis in a prospective cohort study (Hart et al., 2009).

Linoleic acid has also been reported to be the substrate for CYP450 enzymes, including CYP2J2, CYP2C8, CYP2C9, and CYP1A1, leading to the formation of linoleic epoxides 9,10-epoxyoctadecenoic acid (9,10-EpOME), and 12,13-epoxyoctadecenoic acid (12,13-EpOME) known as leukotoxin and isoleukotoxin (Hildreth et al., 2020); these epoxides are then metabolized by the soluble epoxide hydrolases (sEH) into the dihydroxyderivatives 9,10-DiHOME and 12,13-DiHOME. The concentration of these compounds is dependent on both the regulation of biosynthetic pathways (CYP450s and s EH) and the dietary intake of their parent fatty acid LA. 9,10-EpOME is a major contributor to pulmonary toxicity in Acute Respiratory Distress Syndrome (ARDS), an effect that is enabled by the conversion into DiHOME (Zheng et al., 2001). DiHOMEs may also play a dual role in inflammation, stimulating neutrophil chemotaxis at low concentrations while inhibiting neutrophil respiratory burst at higher concentrations (Thompson and Hammock, 2007).

### Alpha-Linolenic Acid and Its Metabolites

Just like LA, ALA is defined as an essential fatty acid in mammals (Das, 2006), and its principal diet sources are nuts, fish, leafy vegetables, and seed oils. After absorption, it can be catabolized into longer chain and more unsaturated FAs such as EPA and DHA, but similar to LA conversion into AA within the ω6 series, the endogenous production of ALA derivatives is low in humans. Tracer studies observed conversion of ALA to EPA, ω3 docosapentaenoic acid (DPA), and DHA, from birth to adulthood, in male and female, but in infants the conversion of ALA to DHA is about 1% whereas in adults is even lower (Brenna et al., 2009). ALA supplementation (by diet, capsules etc.) generally increases EPA and DPA, but has limited effects on DHA levels in plasma fractions and in circulating blood cells, while DHA supplementation increases the concentrations of this FA in blood and tissues (Arterburn et al., 2006). It should be also underlined that there is a competition between ω3 and ω6 FAs for the same metabolic pathway enzymes (desaturases and elongases), and that an increased LA intake decreases ω3 LC-PUFAs. Indeed, dietary ALA conversion appears to be decreased by high LA/ALA dietary ratios (Brenna et al., 2009).

Inconsistent data have recently been reported from studies examining how fatty acids, and particularly ω3 LC-PUFAs, can prevent or treat food allergy, atopic dermatitis, and asthma (Venter et al., 2019). These results can be partially justified by differences in bioavailability and interindividual variability in response to supplementation. Providing preformed ω3 derivatives or foods rich in ω3 LC-PUFAs seems more effective than supplying ALA because of the reported limited conversion capability in humans.

Non enzymatic oxidation of ALA originates analogs of isoprostanes termed phytoprostanes (Galano et al., 2017), possessing antinflammatory activities (Gilles et al., 2009). ALA-enriched diet in rats was reported to reduce oxidative stress and inflammation during myocardial infarction, while increasing the formation of phytoprostane, suggesting their contribution to the observed effects (Leung et al., 2021). Recent data have also shown that novel metabolites generated by gut lactic acid bacteria from ALA, namely 13-hydroxy-octadecadienoic acid and 13-oxo-octadecadienoic acid, are capable to induce, both *in vitro* and *in vivo*, the differentiation of macrophage toward the anti-inflammatory phenotype M2 (Ohue-Kitano et al., 2018), through the activation of G protein-coupled receptor 40.

### Monounsaturated Fatty Acids

Dietary MUFAs sources are both vegetable (e.g., olive oils, nuts, and seeds) and animal (e.g., meat from poultry and pig). The most abundant MUFA within the MD is oleic acid (about 90% of all MUFAs), followed by palmitoleic and vaccenic acids. A recent meta-analysis focusing on the effects of different dietary sources of MUFAs on CVD provided evidence that only olive oil was associated with a significant risk reduction of all-cause mortality, cardiovascular events, and stroke (Schwingshackl and Hoffmann, 2014), consistent with the fact that virgin olive oil is a supplier of other biologically active components such as polyphenols (Visioli and Bernardini, 2011), in particular oleuropein, in addition to MUFA.

### Phenolic Compounds

The beneficial effects of oils have been widely attributed to their content in phenols, and more than 8,000 phenolic compounds have been identified. The structure of polyphenols is characterized by aromatic rings surrounded by hydroxyl groups (Quideau et al., 2011). Among these bioactive oil components there are tocopherols and tocotrienols (corn oil, soybean oil, wheat oil, and others), flavonoids (olive oil, sunflower seed oil), sterols, and phenolic acids (as esterified or free molecules, aldehyde forms, and glycosides). With exception of sterols that have beneficial effect on serum lipids (decreasing LDL-C and increasing HDL-C), the other compounds possess mainly radical scavenging, antioxidant and anti-inflammatory activities (Pandey and Rizvi, 2009), and the ability to modulate the immune response, affecting the multiplication of white blood cells and the production of cytokines (Gorzynik-Debicka et al., 2018). Different components alone have been tested *in vitro*, in cells or cell free assays, and some extra virgin olive oil (EVOO) phenolic compounds, but not all of them, inhibit IL-1β, PGE_2_, and INFγ production (Miles et al., 2005). *In vitro* experimental evidence also showed significant anti-neuroinflammatory effects of lignanamides from hemp seeds (Luo et al., 2017; Wang et al., 2019). In *in vivo* experiments, sesamol decreased oxidative stress and inflammation (Kuhad and Chopra, 2008), and sesamine (0.2% in diet) decreased lipid peroxidation in plasma and liver in rats (Yamashita et al., 2000). Sesame lignans were also investigated in human, comparing the effects of a supplement of sesamin/episesamin 1:1 ratio (10 mg) + vitamin E (101 mg) formulated in wheat germ oil with wheat germ oil alone, showing a significant increase of antioxidant capacity evaluated as an increased lag-time in plasma LDL oxidation (Takemoto et al., 2015).

The predominant compounds found in EVOO are represented by oleuropein, hydroxytyrosol, and their derivatives (Pedan et al., 2019). The European Food Safety Authority (EFSA) approved in 2011 a claim that EVOO’s polyphenols protect blood lipids against oxidative stress at a minimal dose of 5 mg/kg/day. The 5 mg of hydroxytyrosol should be available by consuming 23 g of EVOO in the context of a balanced diet (EFSA Panel on NDA, 2011).

Lipophenols are an emerging class of molecules that have been studied in these last years; they are characterized by condensation (esterification/acylation) of polyphenols and fatty acids, mainly unsaturated ones. Phenols and PUFAs, as described above, are natural compounds both endowed with biological activities on inflammation, oxidation, cancer, and CVDs, and the combination of these molecules could be of therapeutic advantage. In particular, the conjugation of polyphenols, such as flavonoids, phloroglucinol, and catechol derivatives with ω3 LC-PUFAs generates lipophenols (or phenolipids) of interest in which ω3 LC-PUFAs confer hydrophobicity, cell membrane penetration, and bioavailability to phenols, while the latter protect PUFAs from oxidation, possibly promoting their beneficial effects (Crauste et al., 2016).

Lipophenols can be obtained by enzymatic or chemical synthesis, but also from natural sources such as algae and marine species. The biological activity of lipophenols has been assessed in *in vitro* assays, frequently in non-cellular system, and only few studies have been performed *in vivo* in experimental animals. The antioxidant activity is a radical scavenging activity, even if the conjugation with FA frequently causes a decrease in the antioxidant properties of phenol, depending on the type of FA and on the phenol site of esterification (Moine et al., 2021). N-acyl dopamine and N-acyl-vanillylamines derivatives (containing a phenolic moiety), have also been shown to inhibit NO, IL-1β, IL-6, and TNF-α production, an effect that was dependent on the nature of FA with major effect for FA with a ketone group, then PUFA, MUFA, and saturated FA (Dang et al., 2011), suggesting a minor role for the phenolic moiety.

However, it must be stressed that the contribution to human health of all phenolic compounds depends on several factors including the concentration and whole oil composition, the extent of absorption and metabolism and the bioavailability in target tissues (Santangelo et al., 2017).

### Vitamins

Vitamin E (α-tocopherol) and carotenoids are lipophilic antioxidants contained in vegetable oils (e.g., canola, olive, and soybean oil). They are known to decrease serum LDL levels and to prevent their oxidation (Upritchard et al., 2000). A large clinical trial showed a significant increase in the risk of prostate cancer in healthy men upon Vitamin E supplementation (Klein et al., 2011) and there is no specific advice on the intake of vitamin E. Its metabolism is related to vitamin C, vitamin B3, selenium, and glutathione, that all should be included within the diet to reach an optimal effect (Kurutas, 2016).

Vitamin E and carotenoids present in oils play an important role in the protection of PUFAs from oxidation: a pilot study carried out in experimental animals showed that while different preparations of fish oil resulted in similar changes in plasma lipids, a significant increase in plasma lipid peroxidation was observed in the absence of fish oil stabilization with a natural antioxidant mixture rich in α-tocopherol (Engstrm et al., 2009). Carotenoids have been well-characterized for their antioxidant activity *in vitro* (Sandmann, 2019), and recent data have also shown their ability to specifically limit PUFA peroxidation in lipid membranes (Widomska et al., 2019).

Vitamin A is abundant in FO, in the liver and in dairy foods, and has a role in maintaining the immune system functions (Gilbert, 2013). Most effects of vitamin A are exerted by its metabolite, retinoic acid (RA), which through ligation of nuclear receptors controls the transcriptional expression of RA target genes. Within the immune system, RA has a central role in orchestrating immune responses and dendritic cells (DCs), and macrophages seem responsible for its production (Erkelens and Mebius, 2017).

## Edible Plants/Seeds Oils

Edible oils are obtained from seeds, fruits, and pulps of plants, including many herbaceous plants, and comprise major components (such as triacylglycerols) and minor compounds (such as sterols, carotenoids, and tocopherols). They are known to be an essential dietary requirement for humans and may also play a critical role in the economy of several countries (in Tunisia 1.7 million ha are planted with olive tree, producing 4% of the olive oil production worldwide; Jabeur et al., 2014). Given their economic value, adulteration with cheaper oils is a common problem (Salah and Nofal, 2021) sometimes resulting in serious effects on consumers health as in the case of the Spanish oil toxic syndrome (Gelpí et al., 2002). In this section, we will review the results of clinical studies carried out with the different commercially available edible oils.

### Hemp (*Cannabis sativa*) Seed Oil

Hemp seed oil is obtained from *Cannabis sativa*, and is characterized by high PUFAs content and low SFAs amounts (for composition see Table 1), with significant amounts of antioxidant such as tocopherols and phenolic compounds (Smeriglio et al., 2016). While studies in humans using supplementation with hempseed oil are scarce, there are nonetheless positive reports showing effects on clinical symptoms of dermatological diseases (atopic dermatitis; Callaway et al., 2005), as well as reduction of plasma triglycerides and improvement of the ratio total cholesterol/HDL cholesterol (Schwab et al., 2006). Nevertheless, the effects on plasma lipids could not be confirmed in subsequent studies in normal volunteers (Kaul et al., 2008) and in adolescent with hyperlipidemia (Del Bo et al., 2019).

**Table 1:** Components of selected edible oils (unless specified otherwise the amounts reported are per 100 g of oil).

Oil Description	Flaxseed (Pennington, 2002)	Olive (Pennington, 2002)	Canola (Pennington, 2002)	Soybean (Pennington, 2002)	Hempseed (Wang et al., 2019)	Cod liver (Pennington, 2002)	Krill (Xie et al., 2019)
Fatty acids, total saturated (g)	8.976	13.808	7.365	15.65	6.67	22.608	
Fatty acids, total monounsaturated (g)	18.438	72.961	63.276	22.783	13.33	46.711	
Fatty acids, total polyunsaturated (g)	67.849	10.523	28.142	57.74	66.67	22.541	
Choline, total (mg)	0.2	0.3	0.2	0.2			
Vitamin E (alpha-tocopherol) (mg)	0.47	14.35	17.46	8.18			14.74–63.0
Vitamin K (mcg)	9.3	60.2	71.3	183.9			
Calcium (mg)	1	1					1,322
Phosphorus (mg)	1						1,140
Magnesium (mg)							360
Iron (mg)		0.56		0.05			
Zinc (mg)	0.07			0.01			
Potassium (mg)		1					
Sodium (mg)		2					
Vitamin D (μg)						250	
Vitamin A (μg)						30,000	16.4–28.5 mg/100 g
Ω-6/ω-3 ratio					1.71–2.27		
Chlorophylls (μg/g)					0.041–2.64		
Tocopherols (mg/100 g)					100–150		
Carotenoids (μg/g)					0.29–1.73		
10:0 (g)	0.008						
12:0 (g)	0.018						
14:0 (g)	0.077					3.568	
16:0 (g)	5.109	11.29	4.298	10.455		10.63	
18:0 (g)	3.367	1.953	2.087	4.435		2.799	
16:1 (g)	0.06	1.255	0.214	0		8.309	
18:1 (g)	18.316	71.269	61.744	22.55		20.653	
20: 1(g)		0.311	1.317	0.233		10.422	
22:1 (g)	0.031					7.328	
18:2 (g)	14.327	9.762	19.005	50.952		0.935	
18:3 (g)	53.368	0.761	9.137	6.789		0.935	
18:4 (g)						0.935	
20:4 (g)						0.935	
20:5 n-3 (g)						6.898	
22:5 n-3 (g)						0.935	
22:6 n-3 (g)						10.968	
Cholesterol (mg)						570	

### Flaxseed Oil

Flaxseed oil (for composition see Table 1) is a good source of essential FA and contains lignans, cyanogenic glycosides, and cyclic peptides. In spite of the rich content of essential FA, the impact of flaxseed oil on serum lipids is controversial (Pan et al., 2009; Prasad, 2009): a reduction of plasma triglycerides and the improvement of the ratio total cholesterol/HDL cholesterol have been reported (Schwab et al., 2006), but has not been confirmed (Kaul et al., 2008). With respect to potential effects on cardiovascular inflammation, a RCT in healthy abdominally obese adults treated for 8 weeks with flaxseed oil capsules found no modifications in C-reactive protein (CRP), serum amyloid A (SAA), IL-6, and TNF-α (Nelson et al., 2007). On the contrary, 6 weeks of flaxseed oil administration resulted in a significant reduction of CRP (Zhao et al., 2004), an effect confirmed by two additional studies that observed a decrease of CRP, SAA, and IL-6 (Rallidis et al., 2003; Bemelmans et al., 2004).

### Olive Oil and Extra-Virgin Olive Oil

Olive oils (for composition see Table 1) possess many health properties of higher nutritional quality, in particular the EVOO (Martín-Peláez et al., 2013). The majority of benefits are ascribed to minimal constituents in the unsaponifiable fraction, like phenolic compounds, phytosterols, tocopherols, and pigments (Mazzocchi et al., 2019). The effects on human health have been linked to its efficacy in preventing and treating of chronic disease resulting from anti-inflammatory, antioxidant, neuroprotective, and immunomodulatory activities (Carmen Crespo et al., 2018). The phenolic part accounts from 50 to 800 mg/kg and the predominant compound is made-up by oleuropein and its breakdown derivatives, hydroxytyrosol and tyrosol, but additional components contribute to the anti-inflammatory effects of EVOO as shown by the NUTRAOLEUM study: in this double-blind RCT, patients were supplemented with three different virgin olive oils (standard, high in phenolic compounds, and enriched with triterpenes oleanolic and maslinic acids) in a 3-week intervention. Over this period of time, plasma inflammatory biomarkers (IL-8 and TNF-alpha) and DNA oxidation significantly decreased in the group of subjects receiving the functional EVOO enriched with triterpenes (Sanchez-Rodriguez et al., 2019).

### Soybean Oil

Soybean oil is extracted from the seeds of the soybean (for composition see Table 1). Phytosterols present in the oil may be responsible of its reported cholesterol-lowering activity (Zhu et al., 2019). The introduction of ∆6 desaturase from primrose (Primula juliae) and ∆15-desaturase from red bread mold (Neurospora crassa) into the soybean resulted in the production of a soybean oil rich in stearidonic acid (SDA; 18:4, n-3). Together, these two enzymes reduce the amount of linoleic acid by converting it into ALA and γ-linolenic acid, which are in turn ultimately converted into SDA, representing up to 20–30% of total fatty acids in the resulting oil. SDA, unlike ALA, may then be able to raise tissue levels of EPA and possibly DHA in humans, making widely used soybean oil a potential dietary source of this “pro-EPA” fatty acid (Harris, 2012). Indeed, two separate clinical trials showed increased EPA and ω3 index in adult overweight subjects (Harris et al., 2008; Lemke et al., 2010), confirming the ability of SDA to raise EPA plasma levels.

### Canola Oil

Canola oil is obtained from the seeds of different species of Brassica family (for composition see Table 1). *Brassica napus* is known as rapeseed and its oil has a high content of erucic acid, a FA suspected of having pathogenic potential, but as edible oil, canola oil originates from selected Brassica typically showing erucic acid levels below 2%. Lin et al. (2013) reviewed the studies investigating the effects of canola oil, reporting significant reduction in total and LDL cholesterol, increased tocopherol levels, and insulin sensitivity. No effects were observed with respect to lipid peroxidation or susceptibility of LDL to oxidation, but platelets showed decreased ATP secretion and aggregation. Inflammatory markers were not affected by canola oil-based diets while potential linkages between canola oil usage and modifications of cancer risk remain undefined.

Recently, industries manufactured the high-oleic canola oil (HOCO), new canola oil with a modified formulation in fatty acids. HOCO is richer in MUFAs, lower in SFAs, and has a lower ratio of ω6/ω3 fatty acids than standard canola oil. A multicenter RCT provided evidence that a DHA-enriched HOCO improves lipid profiles and lowers CVD risk in abdominal obese subjects (Jones et al., 2014).

## Edible Marine-Derived Oils

### Marine Animal-Derived Oils

It is widely recognized that regularly eating fish decreases the risk of CVDs and related mortality (Hu et al., 2002; He et al., 2004), and dietary guidelines for ω3 LC-PUFAs and fish intake recommend two portions of fatty fish per week to assume 250–500 mg/die of EPA + DHA and prevent coronary diseases (American Heart Association Nutrition Committee et al., 2006; Mozaffarian and Rimm, 2006). While a food-based approach is preferable (Kris-Etherton et al., 2007), nutritional supplements are suitable substitutes for people who do not eat fish, and up to 3 g/day of fish oil is “generally recognized as safe” by the United States Food and Drug Administration. Cod liver oil (for composition see Table 1) is a dietary supplement extracted from Atlantic cod containing saturated, monounsaturated, and various PUFAs, including both EPA and DHA, together with vitamin A and D (Hu et al., 2019). While fish oil supplementation is recommended by the American Heart Association for CHD patients, insufficient evidence was found to grant the use in prevention for patient at high CVD risk only (Siscovick et al., 2017). A large meta-analysis of 10 recent RCT also found no effect of marine ω3 PUFAs supplementation on fatal and non-fatal CHD (Aung et al., 2018) fueling the debate about the health benefit of fish oil supplementation, but a new meta-analysis that included also three recent, large RCTs (VITAL, ASCEND, and REDUCE-IT), finally confirmed that marine ω3 supplementation reduce the risk for myocardial infarction, CHD and CVD death, also defining a clear dose-response relationship between the ω3 dose assumed and the effects (Hu et al., 2019).

In consideration of fish being a limited resource, attention to different sources of ω3 LC-PUFAs is also emerging. Krill oil, for example, is obtained from “*Euphausia superba*” and, along with a wide variety of fatty acid compounds (for composition see Table 1), also provides antioxidants, such as the carotenoid astaxanthin, vitamin E, and vitamin A. A RCT in healthy individuals showed a significant increase in EPA and EPA + DHA levels in plasma after consumption of 3 g/die for 4 weeks of krill oil when compared to FO (Ramprasath et al., 2013). Since both FO and krill oils deliver the same amount of total ω3 PUFAs, the results of this study may suggest that the bioavailability of ω3 PUFAs from krill oil is better than that from FO, but a reexamination of the bioavailability studies failed to confirm a difference between FO and krill oil (Salem and Kuratko, 2014). Lipids from different marine sources show a wide variability in ω3 LC-FAs content (Schuchardt and Hahn, 2013), and their bioavailability depends on several factors, including the concomitant intake of food (mainly its fat content) and the co-presence of other components (Kutzner et al., 2017). The marine sources of ω3 LC-FA not only differ in terms of absolute amounts of specific ω3 LC-FA, but also with respect to their chemical structures. In fish and in fish-derived oils, ω3 LC-FA is present primarily as triglycerides and, to a lesser extent, as free fatty acids. In krill oil, besides the two fractions mentioned, a substantial percentage of ω3 LC-FA is bound into phospholipids, raising the possibility that this form of ω3 LC-FA may also affect the bioavailability (Kutzner et al., 2017). It must be noted that in the near future, the capture of krill may also be restricted because of ecological concerns.

### Algae

Algae consist of an intricate and non-specialized cluster of organisms characterized by an elementary reproductive structure and of photosynthetic nature. Currently, many species are cited in the literature as sources of bioactive compounds that are suitable as functional food ingredients (Ibañez and Cifuentes, 2013; Rengasamy et al., 2020). Micro and macro-algae represent a more sustainable source of PUFA-rich oils than fish. The PUFA profile varies among algal species: in macroalgae, lipid content is ~2–5% of dry weight, but the PUFA proportion of these lipids can represent up to 70% (Cofrades et al., 2010). Certain species of microalgae are capable of *de novo* production of LC-PUFAs thanks to their specific enzymatic systems, and the LC-PUFA content varies among species, but EPA and DHA are predominant in most species (Ratledge, 2004).

Algal biomass contains significant amounts of lipid-soluble carotenoids, with fucoxhantin and astaxanthin being the most abundant (Rengasamy et al., 2020). Limited evidence about their activites are available (Šimat et al., 2020), and a meta-analysis of the RCT carried out with astaxhantin showed unclear results (Wu et al., 2020) suggesting that additional studies are necessary to establish their potential health benefits.

The use of marine algae-derived antioxidants and PUFAs is a desirable goal, and in the last 2 decades, the potential of microalgae and microbes as sources of fatty acids has been increasingly recognized, leading to the large-scale production of PUFA supplements (Martins et al., 2013). The process of lipid production from microalgae and other microorganisms, i.e., single cell oil (SCO) production, has been recently proposed, and is of current industrial interest for use of these materials as dietary supplements in adults and infant nutrition (Ratledge, 2004).

## Discussion

Available evidence indicates that consumption of LC-PUFAs, MUFAs, and polyphenols from edible oils correlates to decreased levels of oxidative stress and inflammation. Dietary lipids act directly and indirectly through the formation of oxygenated metabolites possessing potent biological activities, such as eicosanoids and specialized pro-resolving mediators. In consideration of the different and often opposing biological activities of the families of LC-PUFAs oxygenated derivatives, it is of critical importance to assess the relative abundance of their precursors in cell membranes resulting from specific dietary habits, because LC-PUFAs may compete for the same metabolic pathways, affecting the resulting levels of bioactive metabolites in organs and tissues (Zárate et al., 2017). Furthermore the same metabolite sometimes generates opposing effects at different concentrations, as reported for PGE2 that may differentially activate VEGF at low concentrations and IL-8 at higher concentrations (Bonanno et al., 2016), introducing an additional layer of complexity in predicting the final biological outcome resulting from the activation of specific biosynthetic pathways.

The sensibility of LC-PUFAs to peroxidation may also lead to the formation of a number of biologically active metabolites, that in parallel to what observed for enzymatic metabolites possess often opposing biological activities, enhancing inflammation, oxidative stress, and cellular damage on one side, and promoting the resolution of the inflammatory response on the other hand. Recently a web-based interactive interface has been made available to search for thousands of interconnected biochemical pathways leading to specific phenotypes of relevance for the inflammation and its resolution process (Serhan et al., 2020).

The Mediterranean Diet, thanks to its high supply of vegetables, seeds, and marine food sources rich in ω3 lipids, may be considered an anti-inflammatory diet, and the beneficial roles of plant, seeds, and marine-derived oils in the human body are of growing interest. Major consumption of these oils in their present form, or as nutraceutical supplements, as is the case of oils from fish and algae, may highly contribute replacing SFAs with PUFAs in dietary patterns. Nevertheless, the health benefits associated to increased PUFAs concentrations in cellular membranes have been the object of significant debate (Tummala et al., 2019), with the most comprehensive meta-analysis to-date still supporting the efficacy of marine ω3 supplementation in reducing cardiovascular risk (Hu et al., 2019).

In conclusion, it must be noted that the high heterogeneity in oil composition, inclusive of both the fat and the non-fat components, even from the same primary sources, as well the heterogeneity of clinical study designs reporting the beneficial effects of edible oils, may play a significant role in the health outcome associated to their consumption, often making it difficult to propose firm recommendations from both a quantitative and a qualitative standpoint.

## Author Contributions

AM, VC, and PR drafted the manuscript, proofread, and sorted the references. CA and AS critically reviewed and finalized the manuscript, while GM, ST, and M-LS reviewed and edited the manuscript. All authors contributed to the article and approved the submitted version.

### Conflict of Interest

The authors declare that the research was conducted in the absence of any commercial or financial relationships that could be construed as a potential conflict of interest.
